# Von Willebrand Factor, Angiodysplasia and Angiogenesis

**DOI:** 10.4084/MJHID.2013.060

**Published:** 2013-09-02

**Authors:** Anna M. Randi, Mike A. Laffan, Richard D. Starke

**Affiliations:** 1 Cardiovascular Sciences, National Heart and Lung Institute, Faculty of Medicine, Hammersmith Campus, Imperial College London, London, United Kingdom.; 2 Department of Haematology, Hammersmith Campus, Imperial College London, London, United Kingdom.

## Abstract

The large multimeric glycoprotein Von Willebrand factor (VWF) is best known for its role in haemostasis; however in recent years other functions of VWF have been identified, indicating that this protein is involved in multiple vascular processes. We recently described a new role for VWF in controlling angiogenesis, which may have significant clinical implications for patients with Von Willebrand disease (VWD), a genetic or acquired condition caused by the deficiency or dysfunction of VWF. VWD can be associated with angiodysplasia, a condition of degenerative blood vessels often present in the gastrointestinal tract, linked to dysregulated angiogenesis. Angiodysplasia can cause severe intractable bleeding, often refractory to conventional VWD treatments. In this review we summarise the evidence showing that VWF controls angiogenesis, and review the angiogenic pathways which have been implicated in this process. We discuss the possible mechanisms though which VWF regulates angiopoietin-2 (Ang-2) and integrin αvβ3, leading to signalling through vascular endothelial growth factor receptor-2 (VEGFR2), one of the most potent activators of angiogenesis. We also review the evidence that links VWF with angiodysplasia, and how the newly identified function of VWF in controlling angiogenesis may pave the way for the development of novel therapies for the treatment of angiodysplasia in congenital VWD and in acquired conditions such as Heyde syndrome.

## Introduction

The presence of vascular abnormalities in von Willebrand disease (VWD) was first described in the 1960s, when Armand J. Quick, one of the pioneers in the study of coagulation, reported the presence of telangectasias, defined as skin and mucous lesions consisting of dilated small blood vessels that tend to bleed (rev in^1^). Since then, several groups have reported the presence of vascular malformation in VWD patients in various localizations, including nail bed,^2^ skin, prostate and most frequently angiodysplasia of the gastrointestinal tract.^3^ These lesions can be responsible for severe, intractable bleeding which is often not responsive to VWF replacement therapy and thus represent a significant unmet clinical challenge. Until recently, the pathological mechanism underlying vascular malformations in VWD was unexplained. However the recent discovery that von Willebrand factor (VWF) regulates blood vessel formation^4^ has shed new light on this syndrome and opened new avenues for the treatment of angiodysplasia. In this review we will summarise the process that led to this discovery, its implications for vascular biology and for the treatment of patients with VWD.

## The Cellular and Molecular Basis of Angiogenesis

Angiogenesis (the formation of new blood vessels from pre-existing ones) is a complex process which involves a cascade of events that require fine spatial and temporal coordination (rev in^5^). The initial pro-angiogenic stimulus, often a growth factor produced in response to hypoxia, activates selected endothelial cells (EC) in the pre-existing vascular plexus to undergo changes in polarity and cytoskeletal remodelling, inducing migration towards the source of the pro-angiogenic stimulus. These cells, named tip cells, maintain contact with the adjacent EC, called stalk cells, which acquire a different phenotype.^6^ Stalk cells proliferate to support the elongation of the new sprout. Eventually tip cells come into contact with other tip cells and through their thin finger-like protrusions (filopodia) engage in a cell fusion process, which is facilitated by tissue macrophages.^7^ Blood flow eventually completes canalisation of the new vascular sprout (rev in^8^). In order to become functional, blood vessels undergo stabilization and maturation, with active remodelling of the newly formed network, recruitment of mural cells and deposition of extracellular matrix.^9^ The process requires coordination between EC and other vascular cells, in particular pericytes and smooth muscle cells.

### Growth factors driving the initiation of angiogenesis: Vascular endothelial growth factor (VEGF)

A large and growing number of molecules involved in regulating angiogenesis have been identified. Some are crucial for the initiation and/or progression of the process and their deficiency or dysregulation is incompatible with vascular development. Many other regulators, however, contribute to downstream steps in this complex process; their defect may give rise to dysfunctional vessels rather than complete disruption of the vasculature (rev in^5^,^10^). The best characterised pro-angiogenic endothelial growth factor is vascular endothelial growth factor (VEGF), a major regulator of vasculogenesis and physiological angiogenesis during embryogenesis, as well as physiological and pathological angiogenesis in the adult (rev in^5^,^11^). The VEGF system is also required for lymphangiogenesis (rev in^12^). VEGF-A is the best characterised member of a family which also includes VEGF-B, VEGF-C, VEGF-D and placental-derived growth factor. These bind to the VEGF receptors (R), of which 3 members (VEGF-R1, -R2 and -R3) have been identified. The complexity of the network is further enhanced by splicing and proteolytic cleavage of the ligands (rev in^13^). The main receptor for VEGF in the vascular endothelium is VEGFR2, which is critical for vascular development as well as adult angiogenesis (rev in^14^). VEGF exerts many effects on the vascular endothelium, including promoting proliferation, migration and survival as well as increased permeability (rev in 14). Binding of VEGF-A to VEGF-R2 on EC stimulates dimerization of the receptor and autophosphorylation of specific intracellular tyrosine residues, leading to activation of intracellular signalling cascades, which lead to cell survival, permeability, migration and/or proliferation.^14^ In vivo, VEGF promotes angiogenesis; however overexpression of VEGF leads to the formation of fragile capillaries, with a disrupted structure, reminiscent of angiomas or angiodysplasia.^15^,^16^

### Growth factors controlling quiescence and vascular stability: the Angiopoietins and Tie-2 system

Whilst VEGF controls the early phases of the formation of a new blood vessel, the system most clearly involved in controlling the maturation and stability of new blood vessels is that of Angiopoietins and the Tie-2 receptor. Angiopoietin (Ang)-1 is produced by non-EC, such as pericytes and mural cells that contribute to vascular stability. Ang-1 binds to the tyrosine kinase receptor Tie-2, which is mainly expressed on EC; Ang-1 signalling through Tie2 receptor promotes survival, quiescence and stability of blood vessels. Ang-1 also has anti-permeability and anti-inflammatory functions (rev in^17^). As ever, the picture is complicated by the fact that in some experimental models Ang-1 has been shown to promote cell migration and angiogenesis, in apparent conflict with its pro-quiescence properties. An interesting model has been put forward which proposes that differences in the localization of Tie-2 receptors on EC and their cell surface partners determines whether this signalling pathway supports quiescence or angiogenesis.^18^,^19^

VEGF and Ang-1 play essential and complementary roles in vascular development and angiogenesis. During embryogenesis, VEGF is required for the formation of the initial vascular plexus, whilst Ang-1 is necessary for the remodelling of this early vascular network into mature blood vessels.^20^ A similar interplay between these two systems seems to take place during adult angiogenesis: both VEGF and Ang-1 are able to promote angiogenesis in vivo;^21^ however VEGF causes vascular permeability and tissue oedema, whilst Ang-1 contributes to the stabilization and the maturation of growing blood vessels.^22^,^23^ Furthermore, Ang-1 administration or overexpression in the dermal compartment can protect from the potentially lethal actions of VEGF as a consequence of uncontrolled plasma leakage.^24^ Co-expression of VEGF and Ang-1 has recently been proposed as a strategy to generate more stable new vessels.^25^

Another crucial regulator of the quiescence/angiogenesis balance is Ang-2. Ang-2 is an antagonistic ligand of Tie-2, which competitively inhibits binding of Ang-1, priming the endothelium for activation and vascular destabilisation. Ang-2 appears to act synergistically with VEGF to promote angiogenesis.^26^ Contrary to Ang-1, Ang-2 is synthesised by EC and stored in organelles called Weibel Palade Bodies (WPB), from where it can be rapidly released upon cellular activation.^27^ So whilst Ang-1 acts as an agonist of Tie-2, promoting structural integrity of blood vessels, Ang-2 acts as a naturally occurring antagonist, promoting vessel destabilisation and growth, as well as inflammation.^28^ Depending on the levels of other growth factors, such as VEGF-A, Ang-2 can also promote vessel regression (rev in^29^). The angiopoietin-Tie-2 system is also an area of intensive research for the development of modulatory drugs (rev in^30^).

### Extracellular cues and cell adhesion receptors controlling angiogenesis: integrin αvβ3

Molecular interactions mediated by several adhesion receptors and signalling complexes between cells need to be coordinated to maintain the integrity of the vessel and ultimately to stabilise the newly formed capillary. The extracellular environment is crucial for physiological development of the nascent sprout interaction; cell surface receptors of the integrin family mediate adhesion to and signalling by the extracellular matrix (ECM). Integrins are heterodimeric transmembrane proteins involved in the interaction of cells with their extracellular environment. In response to extracellular cues, integrins are able to transmit so called “outside-in” signals to the cell leading to the activation of signalling cascades via various pathways including those of cellular adhesion and migration. The extracellular conformation of integrins can also be modulated by intracellular processes and transmit so called “inside-out” signals leading to changes in the way the receptor interacts with its extracellular matrix environment and modulation of protease activity (rev in^31^). One integrin receptor in particular, αvβ3, which is expressed on EC and is the best characterised endothelial receptor for VWF, has been shown to play a crucial role in angiogenesis and is a therapeutic target for cancer. The expression of αvβ3 is up-regulated in tumour associated blood vessels^32^ and drugs targeting αvβ3 have shown some success in clinical trials (rev in^33^); however its role appears quite complex, since deficiency of this integrin in the mouse has been linked with increased VEGFR2-dependent angiogenesis.^34^ Interestingly αvβ3 can associate with VEGFR2 and crosstalk between these receptors can stimulate reciprocal activation (rev in^35^). Ang-1 and -2 have been shown to be able to regulate integrin mediated cell adhesion^36^ and Ang-2 can modulate αvβ3 integrin signalling.^19^,^37^

## Angiodysplasia: Vascular Lesions Linked to Abnormal Angiogenesis

Angiogenesis plays a crucial role during embryonic development and in specific processes during adulthood, such as wound healing and the menstrual cycle. Excessive or insufficient angiogenesis has been linked to a growing number of diseases (rev in^38^), and over the last few decades major progress in the understanding of the cellular and molecular basis of the process has been achieved. In parallel to the scientific progress, there has also been intense drug development activity in the search for inhibitors or activators. The area of vascular malformations, however, has received less attention and the links with the pathways controlling angiogenesis are poorly understood. The term angiodysplasia defines vascular malformation, also named ectasia, which affects submucosal veins, mucosal venules and capillaries. The abnormal vascular plexus is fragile and the architecture is disrupted, with possible arteriovenous communications. Angiodysplastic lesions are most commonly observed in the gastrointestinal (GI) tract and are the most common cause of occult GI bleeding in subjects over 65. A firm diagnosis of angiodysplasia may be difficult to achieve, partly because bleeding may be intermittent and partly because not all lesions are accessible to endoscopy. Although angiodysplasia is most frequently located in the proximal large colon (80% of lesions) which is visible by conventional methods, 15% of lesions are located in the small bowel and these may be either missed or require capsule endoscopy, which is not universally available. However, the use of capsule endoscopy has increased the diagnostic yield in patients with obscure GI bleeding to over 60% and as high as 93% in some series, depending on patient selection. This is a significant improvement over push enteroscopy, but in a small number of cases the diagnosis is one of exclusion based on the clinical picture of recurrent GI blood loss.^39^

Despite the limited number of studies on the cellular and molecular basis of angiodysplasia, a link between angiodysplastic lesions and angiogenesis has been identified. The expression of the angiogenic growth factors VEGF and bFGF was found to be increased in samples of angiodysplastic tissue isolated from patients presenting with angiodysplasia.^40^,^41^ Also, increased plasma levels of VEGF have been reported in patients with hereditary haemorrhagic telangectasia (HHT), who present with multiple angiodysplastic lesions,^42^ and patients with genetic or acquired VWD^43^ (see below).

## Von Willebrand Factor as a new Regulator of Angiogenesis

Von Willebrand factor (VWF) is a large multimeric plasma glycoprotein well known for its crucial role in haemostasis, where it mediates platelet adhesion to the endothelium and the sub-endothelial matrix, and acts as a carrier for coagulation factor VIII (FVIII) in plasma. Deficiency or dysfunction of VWF causes von Willebrand disease (VWD), the most common genetic bleeding disorder in man.

VWF is produced by EC and megakaryocytes; in EC, VWF can be constitutively secreted or stored in intracellular organelles called WPB, from where it can be secreted in response to various stimuli (rev in^44^). Although platelets contain VWF, plasma VWF levels have been shown to depend almost entirely on VWF from endothelial cells.^45^ The pathways of VWF synthesis, storage and secretion have been extensively investigated (rev in^46^). VWF drives the formation of WPB, which contain numerous proteins (rev in^47^). A proteomic approach has recently identified more WPB proteins.^48^ The list of known and newly discovered WPB molecules, shown in table 1, includes several molecules which play a role in angiogenesis.^47^–^50^ Because VWF is essential for WPB formation, these proteins are dependent on VWF for their storage and regulated secretion (see below).

In recent years, it has become evident that VWF plays multiple roles in the vasculature. VWF has been shown to control smooth muscle cell proliferation, vascular inflammation, cell death and tumour metastasis (rev in^51^). The large, complex structure of VWF protein supports multiple interactions with cell surface receptors and extracellular matrix proteins; in a recent review by Lenting et al,^51^ VWF has been described as a “molecular bus”, which can interact with 20 other partners. The list of VWF interacting molecules is likely to expand, and with this the understanding of its multiple complex functions.

Recently, our group demonstrated a novel function for VWF in the control of blood vessel formation.^4^ Inhibition of VWF expression in EC *in vitro* was found to cause an increase in proliferation, migration and tube formation, all assays related to angiogenesis. Importantly, these findings were replicated in EC from patients with type 1 or type 2 VWD, which were isolated through a novel technique that uses circulating endothelial progenitors expanded in culture. These cells, called blood outgrowth endothelial cells or BOEC, have allowed for the first time access to EC from the patients, thus opening a new window on the cellular mechanisms controlling VWD. In line with these findings, both vascular development and adult angiogenesis were found to be increased *in vivo*, in VWF deficient mice. The mechanism of action of VWF in the control of angiogenesis involves enhanced signalling from the growth factor receptor VEGFR2, since an inhibitor to VEGFR2 restored in vitro migration^4^ and proliferation (Starke, Randi et al, in preparation) to normal. More recently, a similar result was observed following ablation of VEGFR2 expression in EC in vitro by silencing RNA (Starke, Randi et al, in preparation).

How does VWF control VEGFR2 signalling? The data indicate that this may occur through multiple mechanisms (Figure 2 and^4^). VWF was found to regulate two pathways, possibly linked, which may be controlling angiogenesis: an extracellular pathway involving integrin αvβ3 and an intracellular pathway involving Ang-2 storage in WPB. Both these pathways have been shown to influence VEGF signalling.^28^,^34^

Integrin αvβ3 is the main endothelial receptor for VWF.^52^ αvβ3 is clearly implicated in angiogenesis, although there is some controversy as to its exact role. As discussed above, αvβ3 has been shown to both promote^53^,^54^ and repress angiogenesis.^34^ It is likely that the role of αvβ3 on the angiogenic process may depend on the cellular and extracellular context, interacting partners and/or the phase of angiogenesis (rev in^55^). Thus VWF may be modulating angiogenesis partly through interaction with αvβ3 on the endothelial cell surface. Interestingly, αvβ3 levels, function and trafficking were decreased in VWF-deficient EC,^4^ suggesting that VWF may regulate αvβ3 activity in multiple ways.

**Figure 1:**
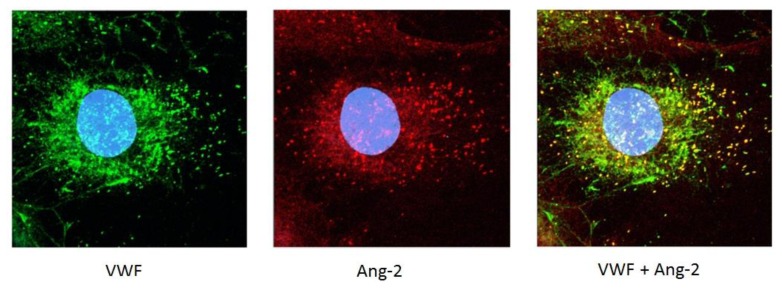
VWF and Angiopoietin-2 (Ang-2) co-localise in Weibel Palade Bodies (WPB) in Human Umbilical Vein Endothelial Cells (HUVEC). WPB are visible as discrete rod-like structures inside the cell. See text for details.

VWF may also control angiogenesis through an intracellular pathway which involves Ang-2. Ang-2 is normally stored WPB with VWF (Figure 1 and^27^). In the absence of VWF, no WPB are formed; therefore Ang-2 may be constitutively released from the cells and presumably acts as a destabilizing, pro-angiogenic agent, as described above. Indeed our studies showed that in VWF-deficient EC *in vitro*, release of Ang-2 was increased.^4^ More recent preliminary data from BOEC confirmed these observations, since Ang-2 release from type 1 and type 3 VWD patients was found to be increased compared to control (Starke, Randi et al, in preparation). Interestingly, Ang-2 has been reported to stimulate the internalisation and degradation of αvβ3^37^, which may link the two pathways controlled by VWF.

Besides Ang-2, VWF interacts with or regulates the storage of several proteins which have been implicated in the control of angiogenesis, including interleukin-8,^50^ galectin-1^56^,^57^ and galectin-3,^57^,^58^ connective tissue growth factor^59^ and insulin-like growth factor binding protein-7.^48^,^60^ Future studies will determine the relative importance of all these pathways in the control of vascular function and angiogenesis by VWF.

**Figure 2:**
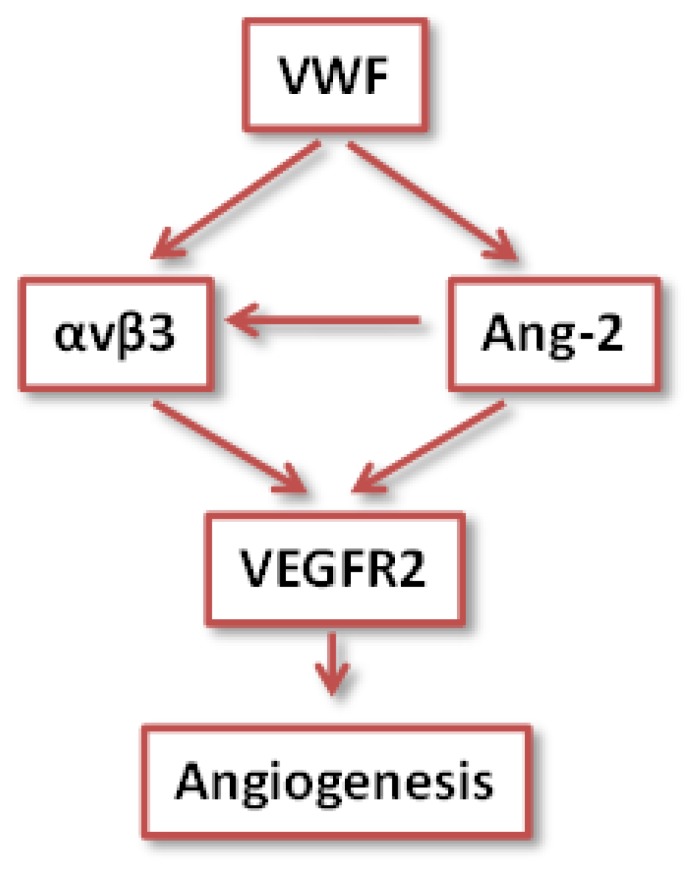
VWF controls angiogenesis through intracellular and extracellular pathways, involving Ang-2 and integrin αvβ3 respectively. These pathways converge to regulate angiogenesis through VEGF Receptor 2 signalling – see text for details.

These studies suggest that VWF controls stability and quiescence through an intracellular pathway, by directing the formation of WPB and hence the storage of Ang-2 (and possibly other angiogenic regulators), and extracellular pathway, by stabilizing αvβ3 on the cell surface and regulating its levels and activity. In the absence of VWF, these pathways are perturbed and result in enhanced VEGF signalling and as a consequence enhanced proliferation, migration and angiogenesis (see model in Figure 2). Interestingly, preliminary data from BOEC from patients with type 1 & 3 vs type 2 VWD suggest that different types may control angiogenesis through different mechanisms, since Ang-2 storage was normal in type 2 VWD patients (Starke, Randi et al, in preparation).

## Von Willebrand Disease, Angiogenesis and Angiodysplasia: Clinical Implications

Many investigators have described an association between VWD and angiodysplasia, particularly in the GI tract (rev in^1^,^61^–^63^); severe GI bleeding, which is often not resolved by conventional treatments, remains one of the most serious unmet clinical needs in VWD. Our data suggest that disturbed angiogenesis is linked to the development of angiodysplastic lesions in these patients. Angiodysplasia is most often observed in VWD patients lacking high molecular weight VWF multimers. The survey carried out by Fressinaud and Meyer reviewed histories from 4503 patients with VWD and found the incidence of angiodysplasia to vary with the VWD subtype. Angiodysplasia was most frequently associated with loss of VWF high molecular weight multimers (HMWM), being found in 2% of type 2 and 4.5% of type 3 respectively. In this study, no angiodysplasia in type 1 VWD was reported. Another study found a particular association with the VWD Type 2A mutation S1506L.^64^ Interestingly, vascular malformations and GI bleeding are also associated with acquired VWD, often in combination with aortic stenosis, in a triad that has been named Heyde syndrome (rev in^65^), which is also associated with loss of VWF HMWM. Heyde syndrome typically responds to aortic valve replacement with restoration of the normal multimer pattern and cessation of bleeding. For many years it was unclear whether this relationship was one of enhanced detection due to low levels of VWF or whether there was a causal relationship between VWF and GI bleeding. The finding that VWF can directly control vascular stability and angiogenesis provides the first mechanistic link and opens the way to possible novel therapeutic approaches to GI bleeding in VWD. So far, no evidence for a specific role of HMWM has been described in the molecular and cellular models in angiogenesis. However the molecular studies have identified both extracellular and intracellular pathways in the control of angiogenesis; thus it is possible that HMWM may affect the interaction of VWF with EC. Future studies will be required to determine the role of VWF multimers in angiogenesis.

Initial treatment of GI blood loss in patients with VWD is logically carried out with VWF replacement therapy, which can reduce the incidence and severity of recurrent bleeding. However, the von Willebrand Disease Prophylaxis Network (VWD PN) study showed that prophylaxis was less successful at reducing GI blood loss than it was in reducing joint bleeding or menorrhagia.^66^ Moreover, it is well recognised that a subgroup of patients continue to have significant blood loss despite otherwise adequate replacement therapy. The failure of VWF replacement coupled with increased understanding of angiogenesis has prompted exploration of alternative therapies for this problem. Some success has been reported with thalidomide in angiodysplasia with or without VWD but this agent has a high incidence of side effects.^67^,^68^ Most recently striking successes have been reported using atorvastatin which has been utilised for its anti-angiogenic effect, but further trials will be required to determine whether this is reproducible.^69^,^70^ Moreover, the characterisation of the molecular pathways through which VWF regulates angiogenesis will provide novel therapeutic targets for the treatment of angiodysplastic GI bleeding.

## Conclusions

The finding that VWF regulates angiogenesis clearly has a number of important implications. Firstly, it provides a novel link between VWD and angiodysplasia, which is likely to have therapeutic implications for the future. Secondly, it points the way to investigating the role of VWF in normal development and healing but also in pathological processes such as tumour growth, all of which depend on angiogenesis. We anticipate that these investigations will lead to novel agents to modulate angiogenesis for therapeutic benefit. A critical question for both of these problems will be determining the relative roles of intra- and extra-cellular VWF in regulation of angiogenesis. We therefore remain some way from translation of these exciting findings into clinical practice. Experience to date suggests that replacement therapy does not always correct the defect in angiodysplasia and it is unlikely that simple infusion of VWF will be a panacea for abnormal vasculature.

## Figures and Tables

**Table 1:** Known and potentially novel WPB content (based on Metcalf et al and van Breevoort et al. 47,48).

78 kDa-regulated protein
α1,3-Fucosyltransferase VI
α-2-HS-glycoprotein
Angiopoietin-2
Biglycan
Calcitonin gene-related peptide
Calreticulin
CD63
Cell Surface glycoprotein MUC18
Clusterin
Collagen alpha-1(I) chain
Collagen alpha-1 (III) chain
EGF-containing fibulin-like extracellular matrix protein 1
Endoplasim
Endothelial protein C receptor
Endothelin-1
Endothelin-converting enzyme
Epididymis-specific alpha-mannosidase
Eotaxin-3
Insulin receptor-related protein
Insulin-like growth factor-binding protein 7
Integrin alpha-5
Interleukin-8
Lysozyme g-like protein 2
Matrix Gla protein
Multimerin-1
Nucleobindin-1
Osteoprotegerin
Pentraxin-related protein PTX3
Plasma alpha-L-fucosidase
Plasma glutamate carboxypeptidase
Plasminogen activator inhibitor 1
Platelet endothelial cell adhesion molecule
Plexin-D1
Protein disulfide-isomerase A3
Protein disulfide-isomerase A4
Protein disulfide-isomerase
P-selectin
Puromycin-sensitive aminopeptidase-like protein
Rab3D
Rab27A
Serpin H1
SPARC
Thrombospondin-1
von Willebrand factor A domain-containing protein 5B1
von Willebrand factor
V-set and immunoglobulin domain-containing protein 8
